# Novel Bacterial Diversity and Fragmented eDNA Identified in Hyperbiofilm-Forming *Pseudomonas aeruginosa* Rugose Small Colony Variant

**DOI:** 10.1016/j.isci.2020.100827

**Published:** 2020-01-09

**Authors:** Binbin Deng, Subhadip Ghatak, Subendu Sarkar, Kanhaiya Singh, Piya Das Ghatak, Shomita S. Mathew-Steiner, Sashwati Roy, Savita Khanna, Daniel J. Wozniak, David W. McComb, Chandan K. Sen

**Affiliations:** 1 Department of Surgery, Indiana Center for Regenerative Medicine and Engineering, Indiana University School of Medicine, Indianapolis, IN 46202, USA; 2 Department of Surgery, The Ohio State University Wexner Medical Center, Columbus, OH 43210, USA; 3 Center for Electron Microscopy and Analysis, College of Engineering, The Ohio State University, Columbus, OH 43212, USA; 4 Departments of Microbial Infection and Immunity, Microbiology, Infectious Disease Institute, Ohio State University, Columbus, OH 43210, USA; 5 Department of Materials Science and Engineering, The Ohio State University, Columbus, OH 43210, USA

**Keywords:** Microbiology, Microbiofilms

## Abstract

*Pseudomonas aeruginosa* biofilms represent a major threat to health care. Rugose small colony variants (RSCV) of *P*. *aeruginosa*, isolated from chronic infections, display hyperbiofilm phenotype. RSCV biofilms are highly resistant to antibiotics and host defenses. This work shows that RSCV biofilm aggregates consist of two distinct bacterial subpopulations that are uniquely organized displaying contrasting physiological characteristics. Compared with that of PAO1, the extracellular polymeric substance of RSCV PAO1Δ*wspF* biofilms presented unique ultrastructural characteristics. Unlike PAO1, PAO1Δ*wspF* released fragmented extracellular DNA (eDNA) from live cells. Fragmented eDNA, thus released, was responsible for resistance of PAO1Δ*wspF* biofilm to disruption by DNaseI. When added to PAO1, such fragmented eDNA enhanced biofilm formation. Disruption of PAO1Δ*wspF* biofilm was achieved by aurine tricarboxylic acid, an inhibitor of DNA-protein interaction. This work provides critical novel insights into the contrasting structural and functional characteristics of a hyperbiofilm-forming clinical bacterial variant relative to its own wild-type strain.

## Highlights

• Hyperbiofilm clinical isolate PAO1*ΔwspF* contain unique cell state and organization
• Bacterial cells in PAO1*ΔwspF* biofilm are morphologically and physiologically unique
• PAO1Δ*wspF*, unlike PAO1 that undergo explosive lysis, release eDNA from live cells
• Aurine tricarboxylic acid, not DNAseI as for PAO1, disrupts PAO1Δ*wspF* biofilm

## Graphical Abstract

**Figure:**
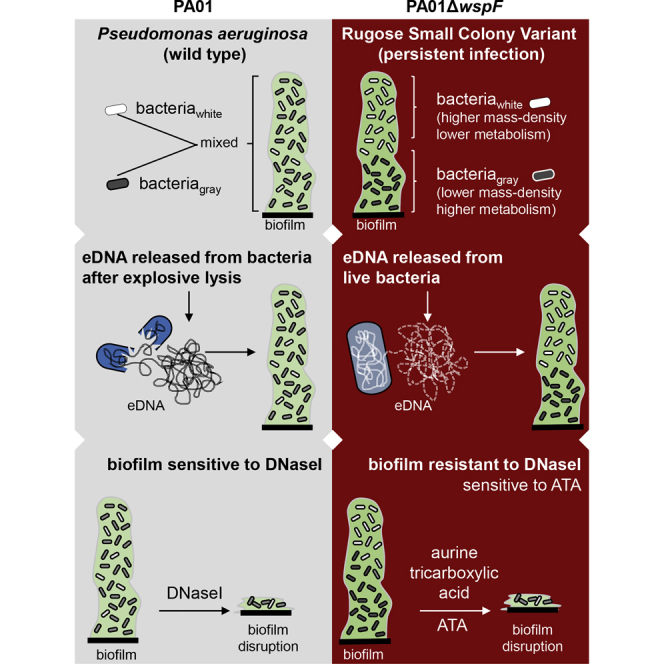

## Introduction

Biofilms are highly resistant to antibiotics and host immune defenses because of their structural and phenotypic characteristics (Høiby et al., 2010, Borlee et al., 2010). Extracellular polymeric substance (EPS) plays pivotal roles in the structural organization of biofilms (Flemming et al., 2016, Gunn et al., 2016). In addition to reinforcing the physical strength of biofilm (Borlee et al., 2010), EPSs also promote microbial interaction and communication (Flemming and Wingender, 2010, Flemming, 2016), enhance horizontal gene transfer (Savage et al., 2013, Merod and Wuertz, 2014), trap nutrients, and even provide nutrients to the persistent bacteria during starvation (Mulcahy et al., 2010). The clinical rugose small colony variant (RSCV) of *Pseudomonas aeruginosa* is hyperactive in biofilm formation during chronic infection (Pestrak et al., 2018, Hauser et al., 2011, Wei et al., 2011). Under laboratory conditions, emergence of some RSCVs relies on loss-of-function mutations in the methylesterase-encoding gene *wspF* (Pu et al., 2018). Such mutations in RSCV result in constitutive overexpression of both Pel and Psl exopolysaccharides (Jennings et al., 2015). RSCVs are difficult to eradicate and are responsible for recurrent or chronic infections (Neut et al., 2007). In biofilms, RSCVs are deeply embedded in self-produced hydrated EPSs (Costerton et al., 1999). The Psl and Pel exopolysaccharides, together with extracellular DNA (eDNA), serve as structural components of the biofilm matrix (Jennings et al., 2015).

The structural characteristics of bacterial biofilm contribute to their pathogenicity (O'Connell et al., 2006). Diversity in the structural elements of bacterial biofilm has been of interest (Donlan, 2002). Insight into biofilm ultrastructure is likely to unveil novel therapeutic strategies for eradicating persistent infection. In this work we sought to investigate the ultrastructure of the hyperbiofilm-producing *P*. *aeruginosa* RSCV strain PAO1Δ*wspF* with reference to its isogenic strain PAO1. Both strains are of direct clinical relevance (Goltermann and Tolker-Nielsen, 2017).

*P. aeruginosa* RSCVs cause persistent infection, because they are recalcitrant to antibiotics and host immune cells (Proctor et al., 2006, Evans, 2015, Pestrak et al., 2018, Wozniak and Parsek, 2014). Scanning transmission electron microscopy (STEM) tomography is powerful in unveiling the structural characteristics with nanometer-scale spatial resolution (Aoyama et al., 2009, Sousa and Leapman, 2012). Insight gained from STEM imaging and tomography has led to novel mechanistic hypothesis. It was thus gleaned that inhibition of EPS protein-eDNA interaction is a specifically effective strategy to dismantling biofilms formed by RSCVs.

## Results

### Distinct Bacterial Phenotype Distribution in PAO1 and PAO1Δ*wsp*F Biofilm

**Figure 1:**
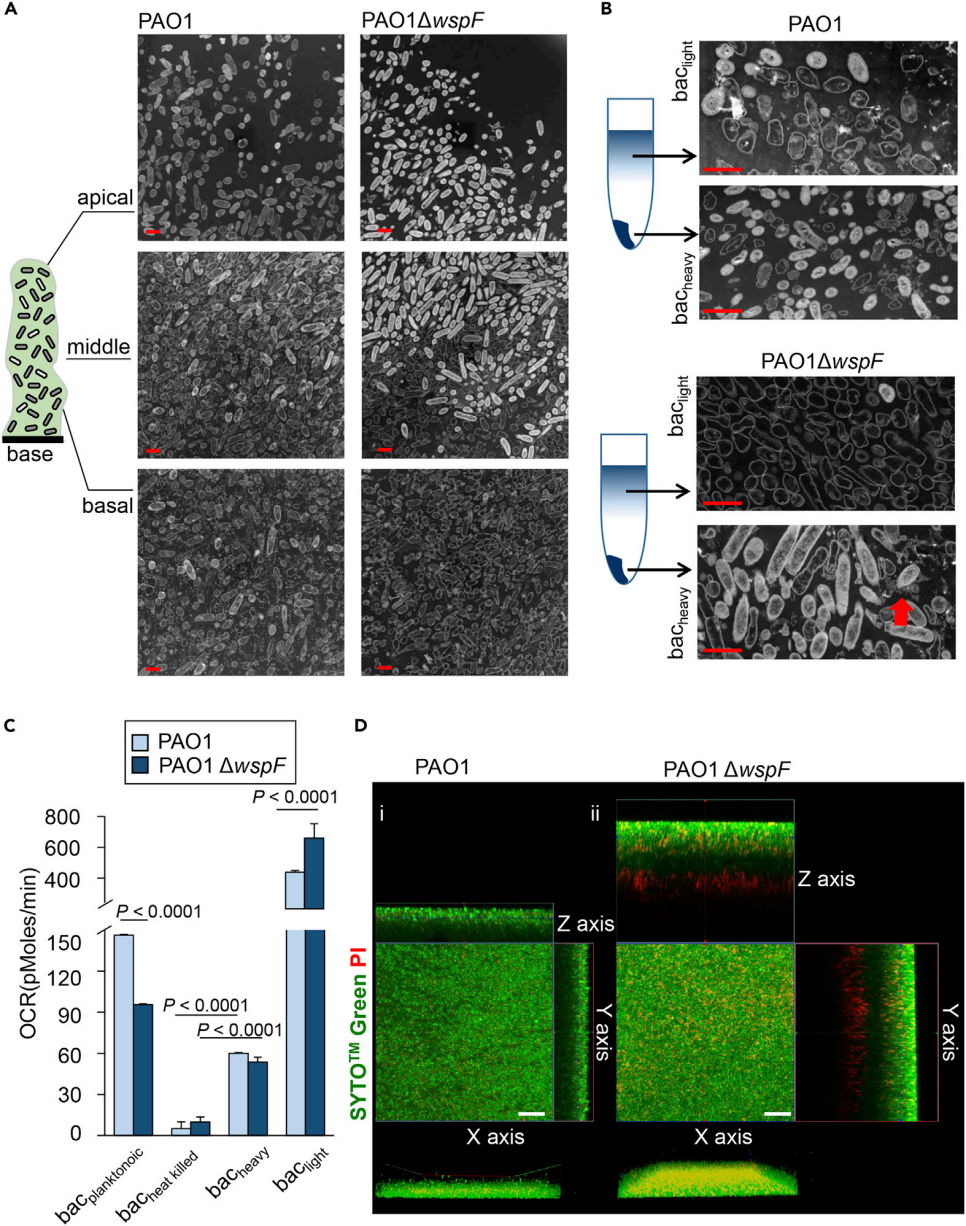
STEM Imaging and Tomography Revealed Distinct Bacterial Phenotype Distribution in PAO1 and PAO1ΔwspF Biofilms (A) STEM images of the *in vitro* PAO1 biofilm and PAO1Δ*wspF* biofilm showed a distinct spatial distribution of two bacterial phenotypes named as bacteria_white_ and bacteria_gray_. Unlike that in PAO1 biofilm where the bacteria_white_ and bacteria_gray_ were homogenously distributed throughout the biofilm, bacteria_white_ and bacteria_gray_ are segregated in the PAO1Δ*wspF* biofilm. Bacteria_white_ was observed from lower-middle to apical area, and bacteria_gray_ was observed from basal to lower-middle area. The pseudo-colored rendering derived from 3D STEM tomographic structure of the PAO1 biofilm and PAO1Δ*wspF* biofilm. Scale bar, 1 μm. (B) STEM images showed the successful separation of bacteria_white_ and bacteria_gray_ in PAO1 biofilm and PAO1Δ*wspF* biofilm. The bac_light_ fraction and bac_heavy_ fraction were obtained from the density gradient centrifugation of biofilm following DNaseI treatment. Scale bar, 1 μm. (C) Real-time changes in oxygen consumption rate (OCR, in picomoles of molecular oxygen per minute) measured on a Seahorse XFe Extracellular Flux Analyzer in bac_light_ fraction and bac_heavy_ fraction of PAO1 and PAO1Δ*wspF* biofilm. (n = 10). Data are mean ± SD. (D) Live dead staining of PAO1 and PAO1Δ*wspF* biofilm using SYTO Green and PI at 48 h. Scale bar, 20 μm. See also Figures S1 and S2.

This work provides insights into the 3D-reconstructed ultrastructure of bacterial biofilm using STEM tomography. STEM imaging and tomography offer the opportunity to investigate the ultrastructure of aggregated macromolecular complexes in the EPS with nanometer-scale spatial resolution. In STEM, a focused electron beam (<1 nm diameter) scans across the specimen and the transmitted signal is collected pixel by pixel. Images collected as a function of sample rotation angle (with respect to the electron beam direction) enable 3D reconstruction (Aoyama et al., 2009, Sousa and Leapman, 2012). In STEM images of non-crystalline materials recorded using a high-angle angular dark field (HAADF) detector, such as the biofilm specimens (Figures 1A and 1B), mass thickness is the dominant contrast mechanism. A region that has higher mass density or is thicker will scatter more electrons. Consequently, the HAADF-STEM signal will be more intense, and the region will exhibit “white” contrast. Unlike conventional confocal microscopy (Figure S1A), STEM imaging of PAO1 and PAO1Δ*wspF* biofilms revealed two distinct subpopulations that were uniquely organized in the hyperbiofilm strain (PAO1Δ*wspF*) compared with that in the wild-type (PAO1) variety (Figures 1A and S1B, Video S1). Two distinct subpopulations, “white” and “grey” contrast, were noted in the STEM-HAADF images (Figure 1A). Henceforth, in this report, these subpopulations are referred to as bacteria_white_ and bacteria_gray_, respectively. In the PAO1 biofilm, bacteria_white_ and bacteria_gray_ were homogenously distributed throughout the biofilm (Figure 1A). In contrast, the PAO1Δ*wspF* biofilm showed a segregated spatial distribution such that bacteria_white_ were found at the apical and bacteria_gray_ at the basal regions of the biofilm (Figure 1A). Thus, bacteria_white_ were localized toward the air interface, whereas bacteria_gray_ were more proximal to the nutrient-supplying basal interface. As the microtomed specimens have negligible variations in thickness, the effect of thickness on the scale of contrast variations can be discounted. Thus the differences between bacteria_white_ and bacteria_gray_ are attributed to their mass-density difference. On the basis of these observations, a density gradient centrifugation approach was developed to separate the two different subpopulations of bacteria: bacteria_white_ and bacteria_gray_ (Figure S2). The pellet obtained after density gradient centrifugation was designated as bac_heavy_ and the supernatant as bac_light_ (Figures 1B and S2). STEM-HAADF images showed that the bac_heavy_ fraction (Figure 1B) was predominantly comprised of bacteria_white_. The bac_light_ fraction was predominantly bacteria_gray_ (Figure 1B). PAO1Δ*wspF* biofilm bacteria were in strict adherence to these rules validating our notion that the bacteria_white_ have higher mass density than the bacteria_gray_. The separation of bacteria_white_ and bacteria_gray_ from PAO1 biofilm cells after density gradient centrifugation was not as efficient as that in the PAO1Δ*wspF* biofilm cells. Although the predominance of bacteria_white_ was indeed more in the bac_heavy_ fraction of PAO1 biofilm, some were present in the bac_light_ fraction as well (Figure 1B).

Video S1. PAO1 and PAO1Δ*wspF* Biofilm, Related to Figure 1A Structural comparison of PAO1 biofilm (pseudo-colored in green) and PAO1Δ*wspF* biofilm (pseudo-colored in gold). Segregation of two bacterial phenotypes was observed in PAO1Δ*wspF* biofilm.

In our effort to investigate functional contrasts between bac_light_ and the bac_heavy_, cellular respiration was studied using a real-time prokaryotic respiration assay (SeaHorse XFe extracellular flux analyzer) (Lobritz et al., 2015). Compared with bac_heavy_, bac_light_ showed elevated oxygen consumption indicative of higher aerobic metabolism of biofilm bacteria localized toward the nutrient interface. Respiration of bac_heavy_ was detected, compared with heat-killed bacteria, indicating that bac_heavy_ were metabolically less active, but not dead (Figure 1C).

In another experimental system studying intact biofilm, the DNA-intercalating dye propidium iodide (PI) stained abundantly toward the air interface in PAO1Δ*wspF* biofilms (Figure 1D). Taken together, PI stain as well as cellular respiration leads to the conclusion that bacteria_white_ have reduced metabolic capacity but have much higher abundance of eDNA in their EPS microenvironment. Thus, this work draws a direct connection between the structural elements and functional properties of bacterial subpopulations within the same biofilm. Importantly, in the hyperbiofilm RSCV, the basal subpopulation proximal to the nutrient interface was metabolically hyperactive compared with the same subpopulation in the wild-type strain (Figure 1C). Such observation may be explained by the finding that in PAO1, the basal hypermetabolic bacteria_gray_ population is somewhat diluted by the presence of few hypometabolic bacteria_white_ cells. However, in PAO1Δ*wspF* biofilm, the basal subpopulation consists of a homogeneous population of hypermetabolic bacteria_gray_ cells.

### PAO1Δ*wspF* Release Segmented eDNA in Biofilm

**Figure 2:**
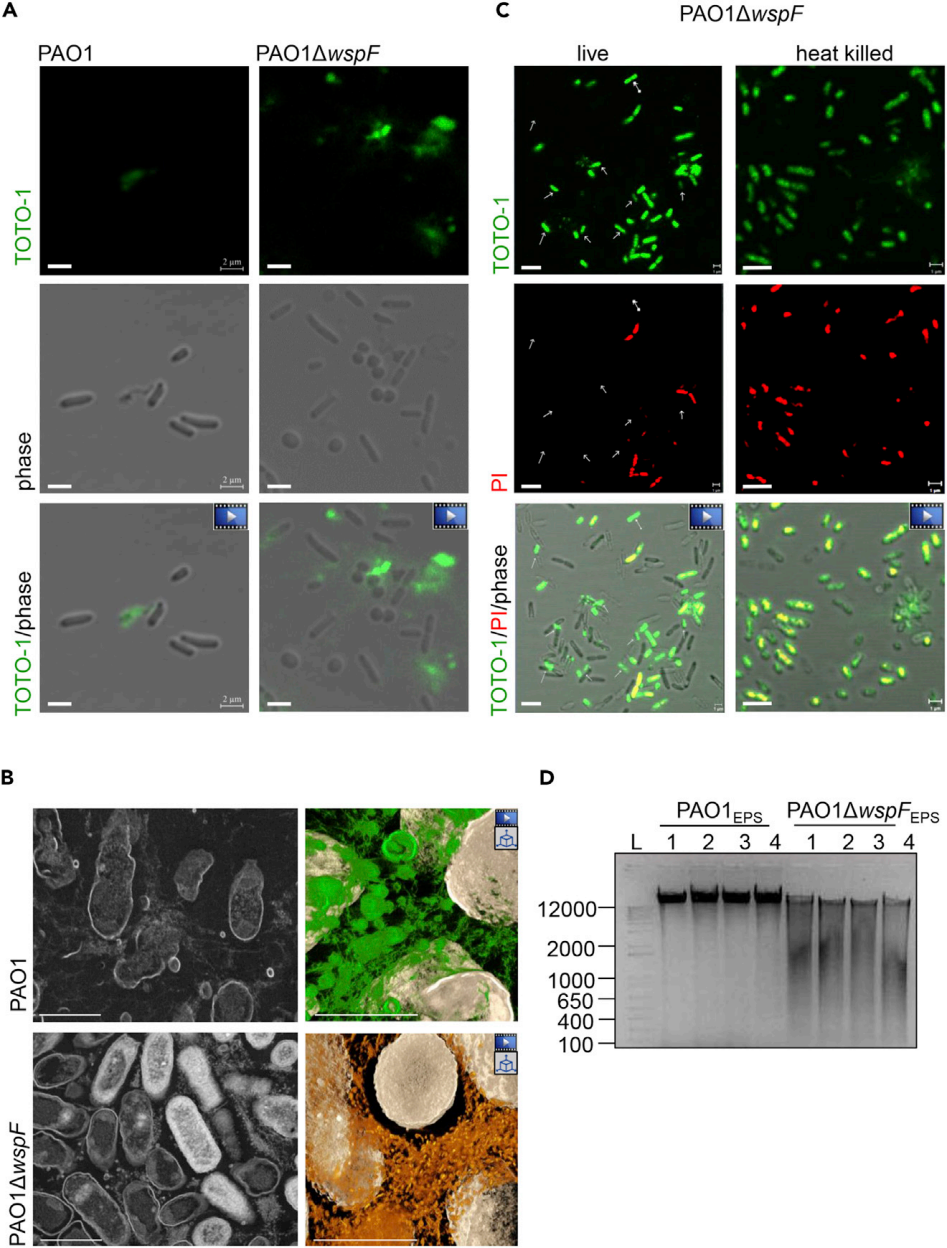
PAO1Δ*wspF* Release Segmented eDNA in Biofilm (A) Confocal microscopic images showing release of eDNA in PAO1 after lysis (left), whereas the PAO1Δ*wspF* showed release of eDNA in intact bacteria (right). Scale bar, 2 μm  Indicate movies in the supplement (Videos S2 and S3). (B) Representative STEM image (top left) showing lysis of PAO1 from 12 different areas of biofilm rich in such fragmented bacteria from middle to basal regions of PAO1 biofilm. 2D rendering of 3D structures (right) of the PAO1 biofilm and PAO1Δ*wspF* biofilm. Cell lysis and release of eDNA (top left) in PAO1 biofilm was observed. Evidence of cell lysis and release of eDNA in PAO1Δ*wspF* biofilm was relatively limited (bottom left). Green and golden pseudo-colors were added in the right images to highlight EPS in PAO1 and PAO1Δ*wspF*, respectively. Movies are available for images with film frame icon (Video S6). Scale bar, 500 nm.  Indicates movies in the supplement.  Indicates 3D reconstruction of the STEM images. (C) Confocal microscopic images showing release of eDNA by live PAO1Δ*wspF*. Scale bar, 2 μm.  Indicates movies in the supplement (Videos S4 and S5). (D) Agarose gel electrophoresis of the DNA isolated from the EPS showed that eDNA is mainly intact in PAO1 biofilm; however, in PAO1Δ*wspF*, eDNA is fragmented. See also Figure S3.

In PAO1, lysis of a subpopulation of bacteria contributes to the eDNA pool, which in turn facilitates the self-organization of biofilm structures (Whitchurch et al., 2002, Turnbull et al., 2016). In our experimental system investigating PAO1, consistent findings were noted. Lysed PAO1 indeed contributed to eDNA as observed from live cell imaging with cell-impermeant DNA-binding dye TOTO-1 that specifically stains eDNA (Figure 2A, Video S2). STEM imaging revealed the products of bacterial lysis within the PAO1 biofilm (Figure 2B top left). In PAO1Δ*wspF* biofilm, however, remnants of lysed bacteria were rarely evident (Figure 2B bottom left). Further investigation into the source of eDNA in EPS of PAO1Δ*wspF* revealed extrusion of DNA from live cells into the extracellular compartment (Figure 2A and Video S3). Such process was not associated with bacterial lysis as reported for PAO1 (Figure 2A). Because PI stains both eDNA and intracellular DNA of bacteria with compromised wall integrity, the PI data from PAO1Δ*wspF* biofilm alone is inadequate to draw any conclusion. To address this, live cell imaging with TOTO-1 and PI was performed in PAO1Δ*wspF* (Figure 2C). Unlike heat-killed PAO1Δ*wspF*, evidence of PI^−^ bacteria showing TOTO-1 staining supports the fact that PAO1Δ*wspF* possess a distinct mechanism of extruding DNA without undergoing lysis as commonly seen in PAO1 (Figure 2C, Videos S4 and S5).

Video S2. Rupture of PAO1 Releases eDNA, Related to Figure 2A The green fluorescence of TOTO-1 is only visible from lysed bacteria. No green fluorescence is observed in intact bacteria.

Video S3. PAO1Δ*wspF* Releases eDNA in the Extracellular Space without Undergoing Lysis, Related to Figure 2A The green fluorescence of TOTO-1 is only visible from intact bacteria.

Video S4. Confocal Microscopic Imaging of Live PAO1Δ*wspF*, Related to Figure 2C The green fluorescence of TOTO-1 is visible from bacteria that did not stain for PI.

Video S5. Confocal Microscopic Imaging of Heat-Killed PAO1Δ*wspF*, Related to Figure 2C The bacteria are non-motile and showing green fluorescence of TOTO-1 that co-localizes with PI.

HAADF-STEM imaging and tomography provides unprecedented insight into the ultrastructure of a wild-type and its corresponding hyperbiofilm variant. In PAO1, heterogeneous mixture of globular debris was abundant in EPS (Figures 2B and S3A, Video S6). In contrast, EPS of PAO1Δ*wspF* biofilm showed thread-like structures associated with vesicular structures (Figures 2B bottom right, S3A right, Video S6). The observed heterogeneous mixture of globular debris in PAO1, which appears white in HAADF-STEM images, was sensitive to DNaseI treatment supporting the notion that it is eDNA (Figure S3C). In PAO1, DNaseI treatment completely eliminated all globular debris-like structures and compromised the structural integrity of the biofilm to a point where fixation of samples for HAADF-STEM imaging was challenging (Figure S3B). In the few cases wherein samples could be processed, distorted morphology of individual PAO1 bacteria were observed (Figure S3C). In cases wherein the structural integrity of the PAO1 biofilm was completely lost, the sloughed off samples were pelleted by centrifugation. Such pellets were processed for STEM imaging as described. Of note, the resulting images provided information on the content of each sample and not on its structure (Figure S3D). Elimination of the globular debris-like structures following DNaseI treatment was evident (Figure S3E). This observation further supports the conclusion that the heterogeneous mixture of globular debris was eDNA. However, unlike the PAO1 biofilm, the PAO1Δ*wspF* biofilm was resistant to DNaseI treatment (Figure S3B). Following DNaseI treatment, PAO1Δ*wspF* biofilm retained appreciable structural integrity including some DNaseI-resistant structures in the EPS (Figure S3C). These retained structures associated with aggregates of vesicular structures only in the EPS of PAO1Δ*wspF* (area pointed by red arrow in Figures S3C, S3F, and S3G). Thus, there are clear differences in the structural characteristics of the biofilm of the wild-type and its variant.

Video S6. EPS in PAO1 and PAO1Δ*wspF* Biofilm, Related to Figure 2B Comparison of the EPS in PAO1 biofilm (pseudo-colored in green) and PAO1Δ*wspF* biofilm (pseudo-colored in gold).

Video S7. STEM Tomography Tilting Series of PAO1Δ*wspF* Biofilm, Related to Figures 1A, 2B, S1B, and Video S1 Tomography tilting series showing the specimen was tilted between −65° and 65° with 1° interval steps. Refer to method “STEM Tomography and Data Processing”.

Video S8. Slice View of Tomogram of PAO1Δ*wspF* Biofilm, Related to Figures 1A, 2B, S1B, and Videos S1 The tomogram reconstructed from the tilt series of Video S13. The viewing area is ~5 μm × 5 μm. Refer to method “STEM Tomography and Data Processing”.

### eDNA in PAO1Δ*wspF* Biofilm Represented Only Part of PAO1Δ*wspF* Genome DNA

**Figure 3:**
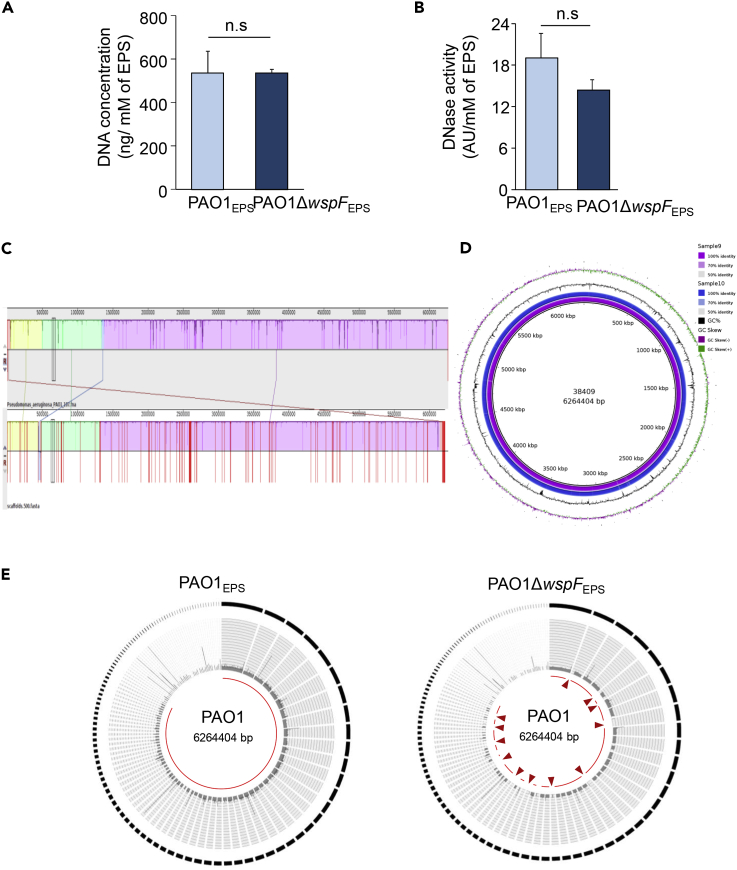
eDNA in PAO1Δ*wspF* Biofilm Was Found to Consist of Only Part of PAO1Δ*wspF* Genome DNA (A and B) Quantification of (A) eDNA and (B) DNase activity from the EPS of PAO1 and PAO1ΔwspF showed no significant difference in the eDNA content and DNase activity. (n = 4) Data are mean ± SD (C) Sorted alignment with PAO1 reference sequence. (D) Circular genome map of PAO1 (accession number: NC_002516) showing the genomic islands (GIs) predicted by IslandViewer and prophages. From the outside: circle 1, GC skew; circle 2, GC content; circle 3, PAO1ΔwspF genome (sample 10); circle 4, PAO1 genome (sample 9). The scale in kilobase pair (kbp) is indicated at the innermost region of the map. (E) Comparison of assembled contigs of PAO1 genomic DNA and PAO1ΔwspF genomic DNA compared with PAO1 reference genome. See also Figure S4.

Explosive lysis of *P*. *aeruginosa* contributes eDNA to EPS of PAO1 (Turnbull et al., 2016). Thus, whole-genomic DNA was expected in the EPS of a PAO1 biofilm (Allesen-Holm et al., 2006). Interestingly, abundance of eDNA in the biofilm of PAO1 and PAO1Δ*wspF* was comparable (Figures 3A and 3B). Our findings on PAO1, the wild-type reference strain of this study, showed that indeed the eDNA of PAO1 biofilm was intact and represented the entire genome (Figures 2D and 3C–3E). Compared with PAO1 biofilm, eDNA of PAO1Δ*wspF* biofilm was mostly fragmented (Figures 2D and 3E) with size range of 25–400 bp (Figure S4). In the context of evidence on DNA extrusion from live PAO1Δ*wspF* bacteria and lack of entire genome representation in the eDNA (Figures 3C–3E) it is concluded that these hyperbiofilm bacteria are capable of contributing eDNA to the extracellular compartment without necessarily having to go through the suicidal path of explosive lysis. In this process, abundant eDNA is deposited as needed for biofilm structure. In the context of hyperbiofilm PAO1Δ*wspF* bacteria, an important question that arises is whether the eDNA is fragmented within the cell and then exported or whether intact DNA exported by the live cell undergoes fragmentation in the extracellular space. In the current work, next-generation sequencing of eDNA from the PAO1 biofilm was identical to that from the PAO1 genome (Figure 3E), supporting the previously reported observation of explosive lysis of PAO1 (Turnbull et al., 2016). PAO1*ΔwspF* biofilm did not follow that pattern. In this case, the eDNA showed little resemblance to the PAO1 genome (Figure 3E). This observation becomes even more interesting considering the fact that both total eDNA content and DNase activity were comparable in the EPS of PAO1 and PAO1Δ*wspF* biofilms (Figures 3A and 3B). These observations led us to test the hypothesis that unlike PAO1, hyperbiofilm-forming PAO1Δ*wspF* bacteria possess the unique ability to extrude DNA fragments as part of bolstering their biofilm structure.

### Interaction of Fragmented DNA with EPS Protein Results in Formation of Robust Biofilm

**Figure 4:**
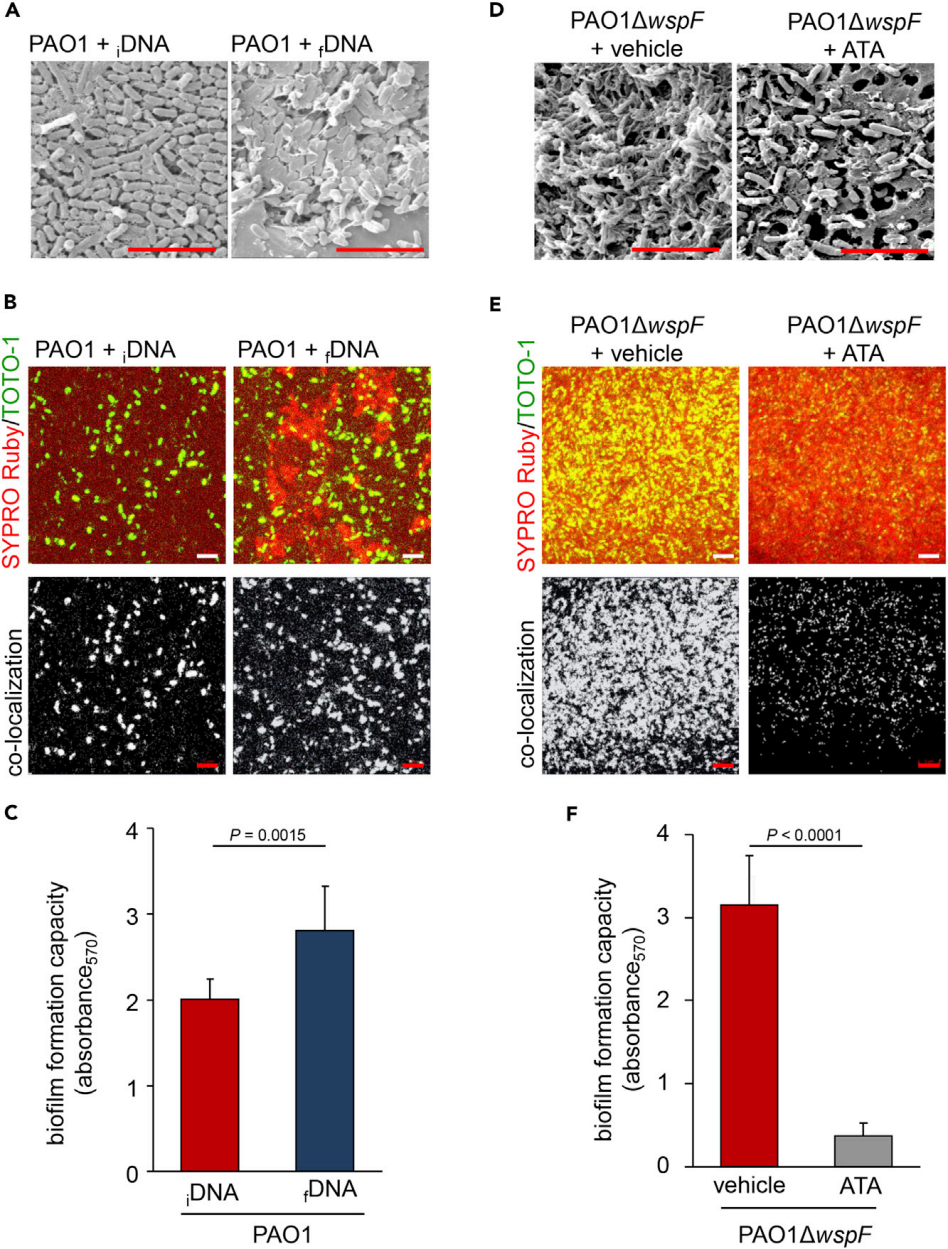
Interaction of Fragmented DNA with EPS Protein Results in Formation of Robust Biofilm (A) SEM images of PAO1 biofilm at 12 h treated with 500 ng intact genomic DNA (_i_DNA) and fragmented genomic DNA (_f_DNA). Scale bar, 5 μm. (B) Confocal microscopic images showing SYPRO Ruby (red) and TOTO-1 (green) staining of PAO1 biofilm at 12 h treated with 500 ng intact genomic DNA (_i_DNA) and fragmented genomic DNA (_f_DNA). The co-localization of EPS protein (red) and eDNA (green) is shown as white dots. Scale bar, 5 μm. (C) Crystal violet assay of PAO1 biofilm at 12 h treated with 500 ng intact genomic DNA (_i_DNA), and fragmented genomic DNA (_f_DNA) (n = 8). Data are mean ± SD. (D) SEM images of PAO1Δ*wspF* biofilm at 24 h treated with buffer and ATA. Scale bar, 5 μm. (E) Confocal microscopic images showing SYPRO Ruby (red) and TOTO-1 (green) staining of PAO1Δ*wspF* biofilm at 24 h treated with buffer and ATA. The co-localization of EPS protein (red) and eDNA (green) are shown as white dots. Scale bar, 5 μm. (F) Crystal violet assay of PAO1Δ*wspF* biofilm at 24 h treated with buffer and ATA (n = 8). Inhibition of DNA-protein interaction compromised *in vitro* PAO1Δ*wspF* biofilm formation. Data are mean ± SD. See also Figures S5 and S6.

In the current work, addition of EPS from PAO1Δ*wspF* to PAO1 augmented biofilm formation. However, addition of EPS from PAO1 to PAO1Δ*wspF* did not influence its biofilm-forming ability (Figures S5A and S5B). To elucidate the functional significance of EPS component eDNA in biofilm formation, intact genomic DNA was isolated from PAO1 and subjected to DNaseI digestion (Figure S5C). Addition of this fragmented DNA to PAO1 showed no significant change in bacterial growth curve when compared with addition of intact DNA to PAO1 (Figure S5D). However, such addition of fragmented DNA accelerated biofilm formation in PAO1. Compared to addition of intact DNA, fragmented DNA showed clear enhancement of biofilm formation (Figures 4A and S5E). Most biofilm matrix proteins stain positive with SYPRO Ruby (Ahire et al., 2016). Compared to intact DNA, fragmented eDNA was more effective in interacting with biofilm matrix proteins (Figures 4B and S5F). Consistently, crystal violet assay for biofilm quantification supported the same conclusion demonstrating that fragmented DNA enhanced biofilm formation (Figure 4C). DNA is known to possess adhesive property, which facilitates interaction with other biomolecules to ensure structural integrity of the biofilm (Okshevsky and Meyer, 2015). Observations of the current study lend credence to the notion that fragmented eDNA, as opposed to intact DNA, provides additional advantage to the process of biofilm formation. Interestingly, hyperbiofilm bacteria utilize this edge to their advantage.

Bacteria with hyperbiofilm characteristics employed fragmented eDNA to achieve better interaction with macromolecules in the EPS (Figures 4D–4F). To test the significance of such interaction in biofilm formation, the EPS isolated from PAO1Δ*wspF* biofilm was incubated with aurine tricarboxylic acid (ATA), a pharmacological inhibitor of protein-nucleic acid binding (Gonzalez et al., 1979). ATA significantly compromised the biofilm-forming ability of PAO1 (Figure S6A). Protein-nucleic acid binding played a significant role in biofilm formation by RSCV (Figures 4D–4F and S6B). However, ATA did not affect bacterial growth as evident from PAO1Δ*wspF* growth curve (Figure S6C). Specifically, ATA limited protein-nucleic acid interaction in PAO1Δ*wspF* biofilm (Figures 4F and S6D).

## Discussion

*P. aeruginosa* RSCVs cause persistent infection, because they are recalcitrant to antibiotics and host immune cells (Proctor et al., 2006, Evans, 2015, Pestrak et al., 2018). Structural characteristics of bacterial biofilm contribute to their pathogenicity (O'Connell et al., 2006). This work is the first to compare the biofilm ultrastructure of a parent strain of *P*. *aeruginosa* with an isogenic RSCV. While commonly used confocal laser scanning microscopy or SEM techniques to understand biofilm structure are of value (Azeredo et al., 2017, Lawrence and Neu, 1999, Schlafer and Meyer, 2017), they are somewhat limited in resolution. This work reports the first evidence for the presence and distribution of two distinct bacterial populations, apical bacteria_white_ and basal bacteria_gray_, in the PAO1Δ*wspF* biofilm. The distribution of these two distinct bacterial populations in the PAO1Δ*wspF* biofilm was not only morphological but also physiological.

Findings of this work demonstrate that the oxygen consumption of basal bacteria_gray_ was elevated compared with that of the apical bacteria_white_ population. These data were consistent with the previous report from the spatial distribution of *Escherichia coli* macrocolony biofilms (Serra and Hengge 2014). According to that report, bacteria in the basal region were dividing with minimal ribosomal synthesis, whereas bacteria in the apical region displayed limited cell division yet robust ribosomal synthesis (Serra and Hengge 2014). This work reports the first identification and separation of these two distinct bacterial populations.

A growing body of research now acknowledges the presence of extracellular forms of DNA and their role as important structural components of the biofilm matrix (Bockelmann et al., 2006). Previously, eDNA was thought to result largely from the lysis of cells or release of plasmids. However, seminal studies by Whitchurch et al. showed that eDNA is a major component of the *P*. *aeruginosa* EPS (Whitchurch et al., 2002). Hence, we looked for the composition and origin of eDNA present in the EPS as a variable for biofilm stability in PAO1ΔwspF compared with PAO1. The formation of a biofilm also relies on the structural proteins that provide the three-dimensional architectural integrity and functionality (Hobley et al., 2015). Negatively charged eDNA interacts with positively charged proteins (Dengler et al., 2015) and polysaccharide (Wang et al., 2015, Jennings et al., 2015) to form the structural backbone of the bacterial biofilm. How eDNA stabilizes the *P*. *aeruginosa* biofilm structure and contributes to antimicrobial tolerance remains unclear. This work recognizes the fact that intact bacterial DNA presents itself as eDNA in PAO1 biofilm supporting the contention that such DNA is delivered by bacterial cell lysis. Explosive lysis of *P*. *aeruginosa* has been shown to be responsible for eDNA contents of biofilm (Turnbull et al., 2016). eDNA in *P*. *aeruginosa* is similar to whole-genome DNA (Allesen-Holm et al., 2006). Consistently, our work reports intact eDNA in the PAO1 biofilm. Interestingly, in a PAO1Δ*wspF* biofilm, eDNA was mostly fragmented. Thus, whether the DNA is fragmented in the matrix or processed inside the bacteria emerges as an interesting question. That bacterial cellular DNA may be exported by live cells has been recently shown in *Staphylococcus aureus* (DeFrancesco et al., 2017). Genome-wide screening for genes involved in forming robust *S*. *aureus* biofilms identified gdpP and xdrA that are involved in the release of eDNA (DeFrancesco et al., 2017). Whether, unlike PAO1, viable non-lytic PAO1Δ*wspF* is capable of digesting part of its own DNA and extruding such digest to support the biofilm structure needs further investigation.

Consistent with the notion that eDNA provides critical support to the biofilm structure, DNaseI treatment compromised PAO1 biofilm. In contrast, the structural integrity of PAO1Δ*wspF* biofilm was mostly unaffected by such enzymatic treatment. After DNaseI treatment, although eDNA was removed at the basal region, thread-like eDNA persisted from the middle to the apical region of the PAO1Δ*wspF* biofilm. Emerging studies reveal that interaction between eDNA and other EPS components may stabilize biofilm structure (Schwartz et al., 2016, Hu et al., 2012, Jennings et al., 2015, Das et al., 2013b). For example, pyocyanin, a metabolite of *P*. *aeruginosa*, interacts with eDNA enhancing bacteria cell aggregation (Das et al., 2013a). In *P*. *aeruginosa* biofilm, negatively charged eDNA and positively charged Pel polysaccharide are cross-linked by ionic forces (Jennings et al., 2015). The Psl-eDNA fiber-like structure helps to form the biofilm skeleton in *P*. *aeruginosa* (Wang et al., 2015).

Biofilms are more susceptible to antibiotics after eDNA is removed by DNase (Kaplan et al., 2012, Tetz et al., 2009). Although DNaseI treatment did not dismantle the biofilm structure of PAO1Δ*wspF*, it was helpful in separating bac_light_ and bac_heavy_ cells, pointing toward a potential role of eDNA in the adhesion of these cells. In *P*. *aeruginosa*, addition of eDNA enhances biofilm structure (Yang et al., 2009). On the other hand, addition of excessive eDNA may inhibit planktonic bacteria growth and biofilm formation (Mulcahy et al., 2008). In this work, cell growth of *P*. *aeruginosa* was not altered in the presence of digested DNA at a concentration of 100 ng/mL (Figure S5D). Interestingly, addition of genomic DNA digest increased DNA-protein interaction and accelerated biofilm formation. Indeed, nucleoid-associated proteins are known to connect eDNA strands in *Haemophilus influenzae* biofilm (Goodman et al., 2011). Targeting eDNA-protein interactions disperses *Burkholderia cenocepacia* biofilms (Novotny et al., 2013 ). Proteomic findings of this work revealed the co-existence of higher abundance of nucleic acid-binding protein and fragmented eDNA at the apical bacteria_white_ region. Inhibition of DNA-protein interaction with ATA blunted biofilm formation by PAO1Δ*wspF*.

STEM images reported herein provide unprecedented comparative insight into the structure of prototypical *P*. *aeruginosa* and its isogenic RSCV strain PAO1Δ*wspF*. This work reports the first evidence for the presence and segregated distribution of two distinct bacterial populations, apical bacteria_white_ and basal bacteria_gray_, in the PAO1Δ*wspF* biofilm. These bacteria were not only phenotypically different but also showed difference in oxygen consumption rate. Furthermore, resistance to DNase digestion in RSCV was attributed to the fact that the eDNA in the EPS was fragmented. The strategy to inhibit protein-DNA interaction using ATA was effective in dismantling biofilms formed by RSCV. Taken together, this work provides unprecedented visual cues into the structure of biofilm formed by *P*. *aeruginosa* upholding clear structural as well as functional differences between wild-type and its hyperbiofilm variant.

### Limitations of the Study

Study of the PAO1Δ*wspF* led to the observation of two different bacterial subpopulations displaying distinct spatial organization in biofilm aggregates. Previous studies have reported explosive lysis of wild-type *P*. *aeruginosa* that contributes eDNA to EPS (Turnbull et al., 2016). We have reproduced that observation in PAO1. However, such explosive lysis was not observed predominantly in the PAO1Δ*wspF*. Live cell imaging and NGS data support that hyperbiofilm-forming PAO1Δ*wspF* bacteria possess the unique ability to extrude DNA fragments from living bacteria as part of bolstering its biofilm structure. These novel observations are based on the study of a single strain that was selected because it is a clinical isolate and therefore of relevance to human health care. Although in our observation we have not noted explosive lysis of PAO1Δ*wspF* bacteria, the possibility that different *P*. *aeruginosa* in other habitats may undergo explosive lysis remains open. We acknowledge that our data may be specific to this clinical isolate and that different *P*. *aeruginosa* may behave differently. Results of this work introduce a new paradigm wherein specific details such as aggregate size and organization may vary across different strains.

## Methods

All methods can be found in the accompanying Transparent Methods supplemental file (Videos S7 and S8).

## Supplemental Information

Document S1. Transparent Methods and Figures S1–S6
