# Supplementary material for: Novel Bacterial Diversity and Fragmented eDNA Identified in Hyperbiofilm-Forming Pseudomonas aeruginosa Rugose Small Colony Variant
Source: iScience. 2020 Jan 9;23(2):100827. doi: 10.1016/j.isci.2020.100827 (PMC6997594; doi:10.1016/j.isci.2020.100827)
Supplement: Document S1. Transparent Methods and Figures S1–S6 [file mmc1.pdf]

iScience, Volume *23*

Supplemental Information

Novel Bacterial Diversity and Fragmented eDNA Identified in Hyperbiofilm-Forming *Pseudomonas aeruginosa* Rugose Small Colony Variant

Binbin Deng, Subhadip Ghatak, Subendu Sarkar, Kanhaiya Singh, Piya Das Ghatak, Shomita S. Mathew-Steiner, Sashwati Roy, Savita Khanna, Daniel J. Wozniak, David W. McComb, and Chandan K. Sen

Figure S1

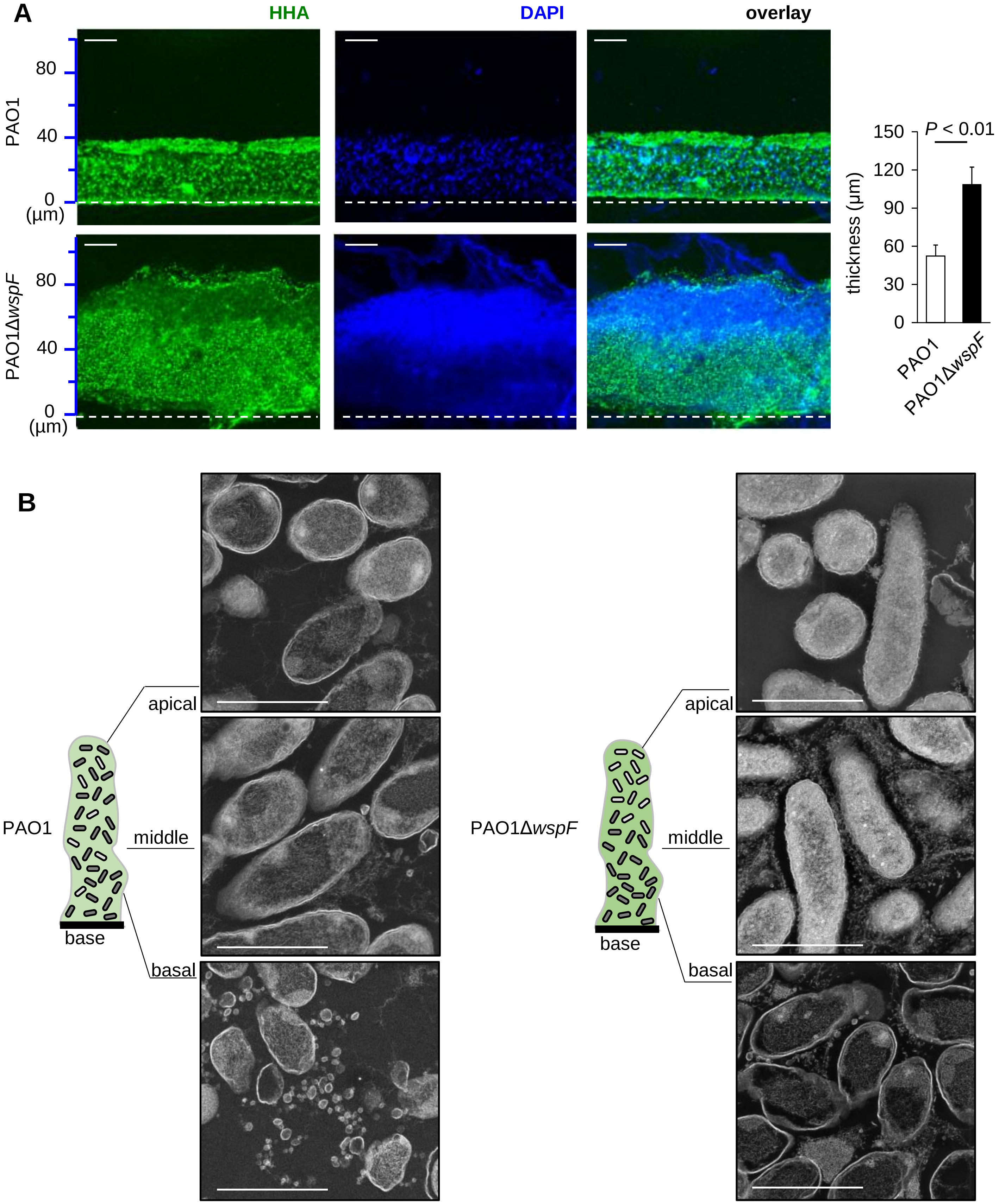

Figure S1. Bacterial segregation in PAO1Δ*wspF* biofilm. Related to Figure 1A. (A) Confocal microscopic images showing the longitudinal section of PAO1 and PAO1Δ*wspF* biofilm after staining with the FITC-HHA lectins (green) and DAPI (blue). Scale bar, 20μm. The white dashed line indicates the PCM membrane. The average thickness of the biofilms are quantified and expressed graphically as mean ± SD. (n=4). (**B**) STEM images showed the biofilms at basal, middle and apical area. Scale bar, 1μm.

Figure S2

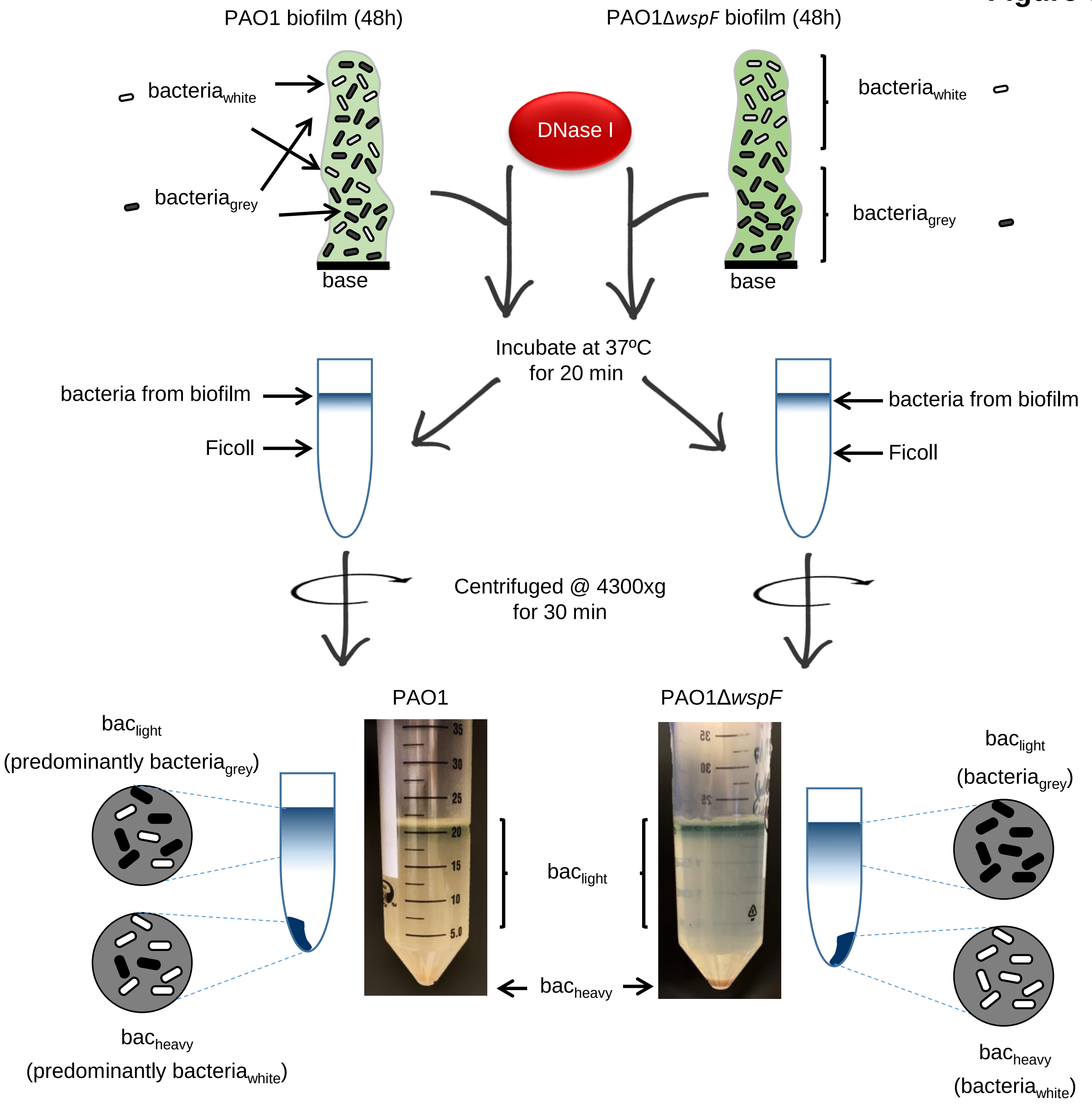

Figure S2. Process of separating bacterial phenotype bacteria$_{white}$ and bacteria$_{grey}$ in PAO1 and PAO1Δ*wspF* biofilm. Related to Figure 1B. Schematic diagram showing the process of separating the two different bacterial phenotype from the biofilm using density gradient centrifugation.

Figure S3

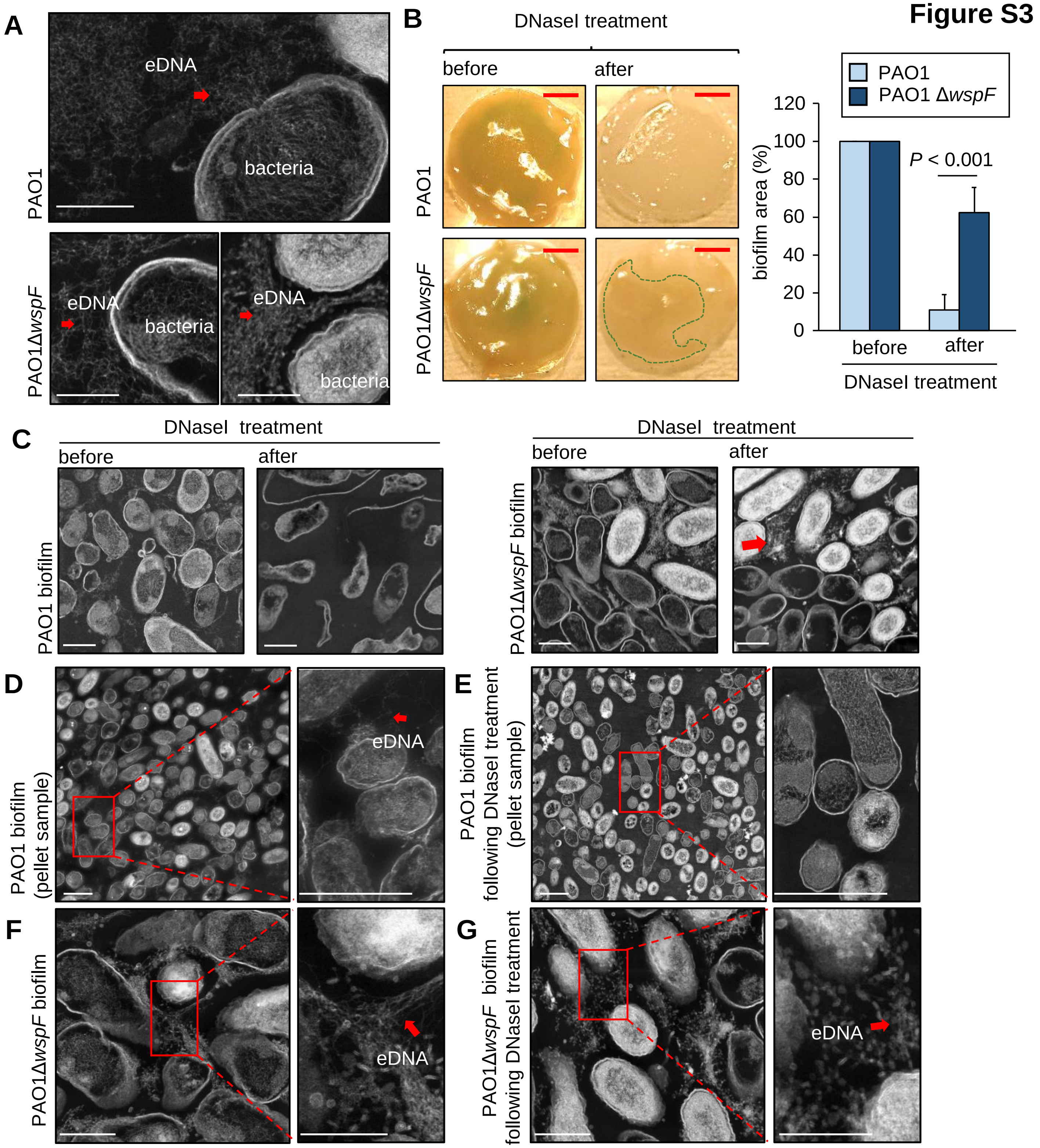

Figure S3. PAO1Δ*wspF* biofilm are resistant to DNaseI digestion. Related to Figure 2. (**A**) eDNA in PAO1 biofilm and PAO1Δ*wspF* biofilm. Upper image showed thread-like eDNA in PAO1 biofilm. Lower left image showed the thread-like eDNA around bacteria$_{grey}$ in PAO1Δ*wspF* biofilm. Lower right image showed the eDNA and cell debris around bacteria$_{white}$ in PAO1Δ*wspF* biofilm. Scale bar, 250nm (**B**) Digital macrophotographs of PAO1 and PAO1Δ*wspF* biofilm before and after DNaseI treatment. The green dotted lines represent the area that remained intact after DNaseI treatment. The area of the biofilm before and after DNaseI treatment were quantified using image J and expressed graphically (n=6). Scale bar, 2.5mm. (**C**) STEM images compared ultrastructure of PAO1 and PAO1ΔwspF biofilm with no treatment and following DNaseI treatment. Although the thread-like EPS largely decreased, PAO1Δ*wspF* biofilm kept structural integrity after DNaseI treatment. Red arrow highlighted unaffected thread-like eDNA at the center of clumps of vesicle-like structure and cell debris (right image). Scale bar, 1μm **(D)** STEM images of pellet sample from PAO1 biofilm showing thread-like structure outside of bacteria. Scale bar, 1μm. **(E)** STEM images of pellet sample form PAO1 biofilm following DNaseI treatment showing no apparent tread-like structure outside of PAO1 bacteria. Scale bar, 1μm. **(F)** STEM images of pellet sample of PAO1Δ*wspF* biofilm without DNaseI treatment. Scale bar, 500nm. **(G)** STEM images of PAO1Δ*wspF* biofilm after DNaseI treatment showing only trace amount of thread-like structure (red arrow) after DNaseI treatment. The inset shows the higher resolution image from the boxed area in the left images. Scale bar, 500nm.

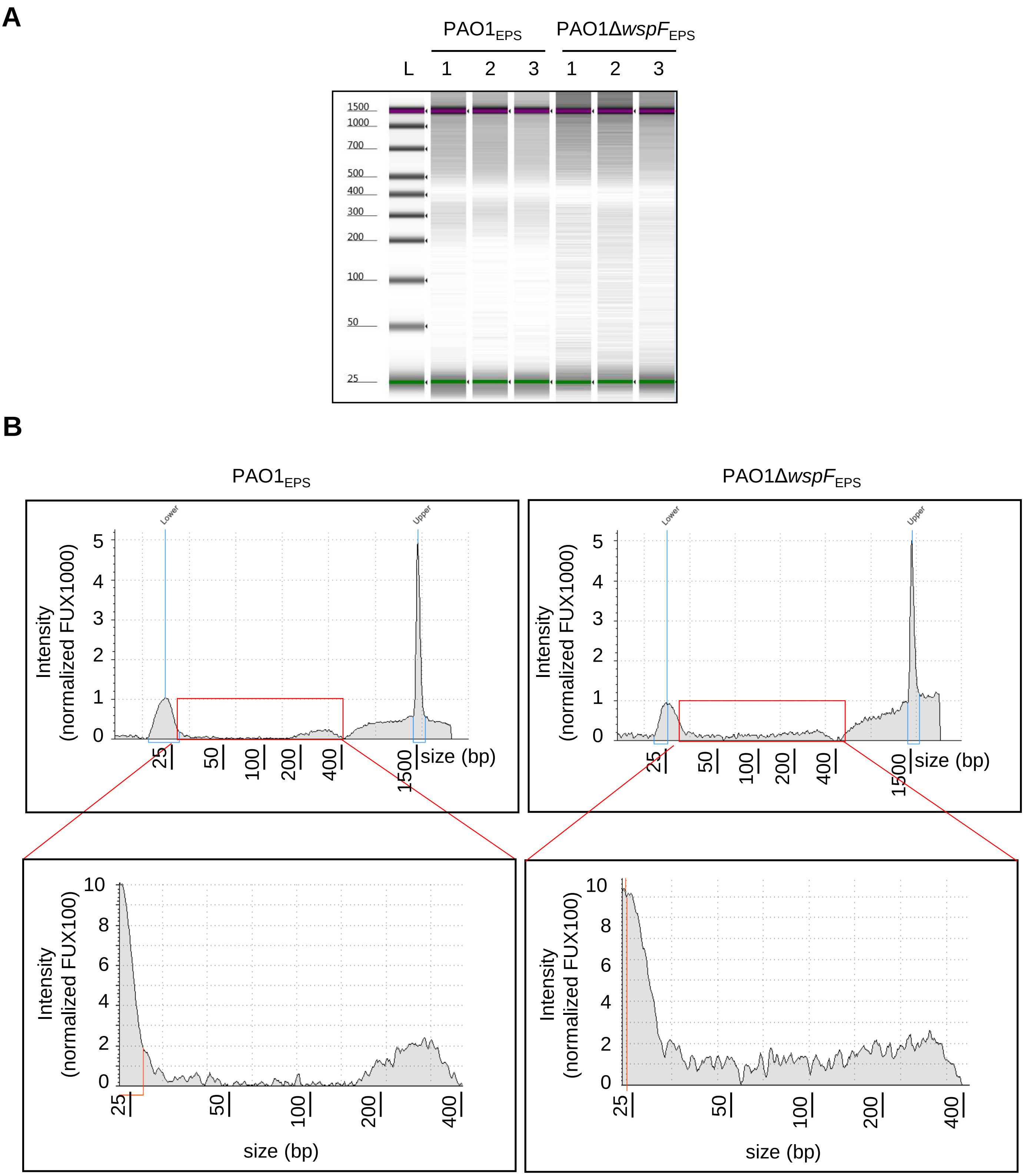

Figure S4. EPRS of eDNA from PAO1 and PAO1Δ*wspF* biofilm. Related to Figure 3. (**A**) Electron plasma resonance spectroscopy (EPRS) of the DNA isolated from the EPS of PAO1 and PAO1ΔwspF, showing higher abundance of fragmented eDNA in PAO1ΔwspF biofilm. The numerical value indicates the sample replicates. (**B**) Histogram of EPRS showing the fragmented DNA in the EPS of PAO1Δ*wspF* biofilm. The region between 400-25 bp were shown as insets.

Figure S5

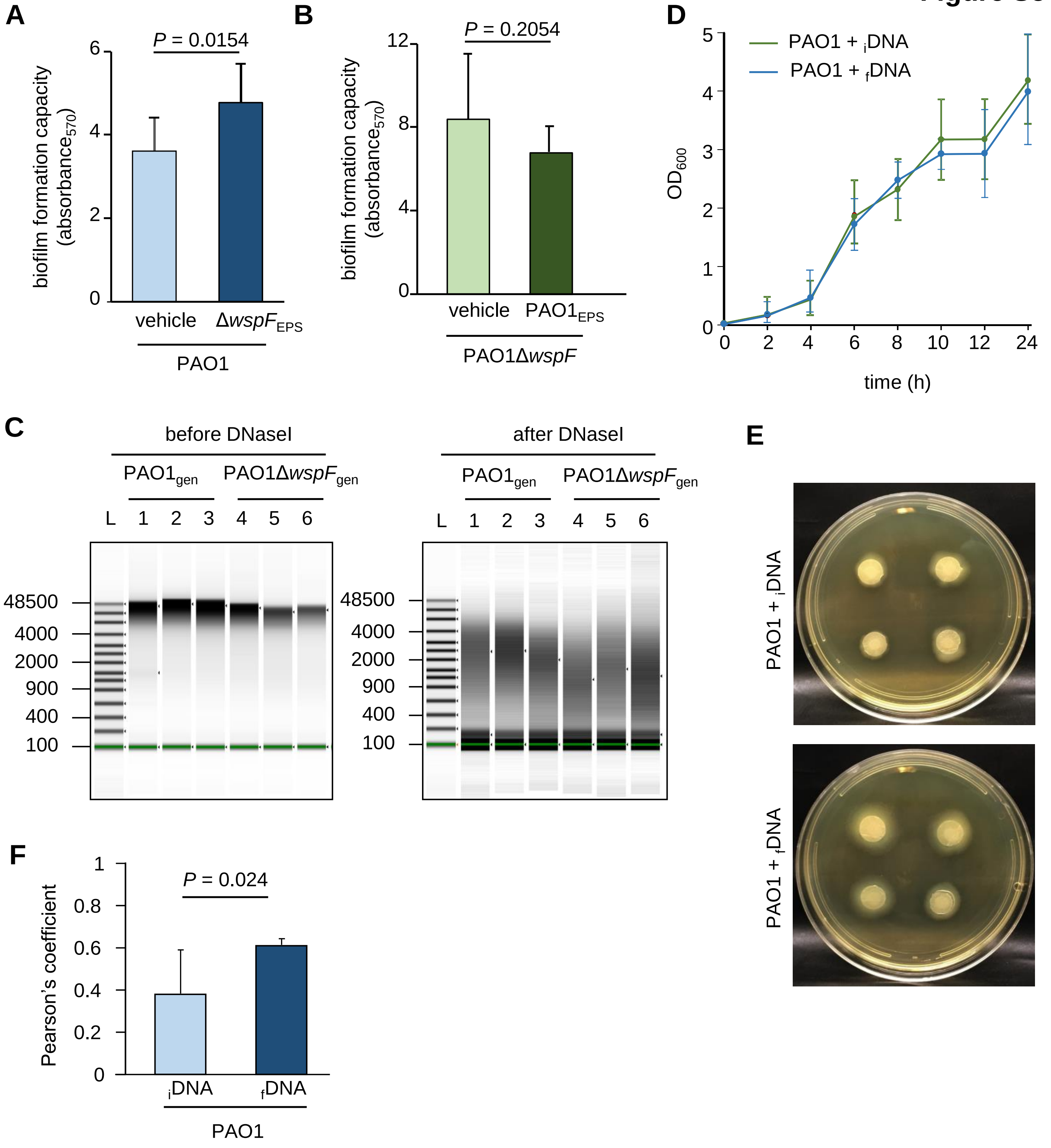

Figure S5. Effect of digested DNA on PAO1 biofilm. Related to Figure 4A-C. (A) Crystal violet assay of PAO1 hydrated biofilm at 12h treated with 1µl of PAO1*ΔwspF* EPS (2ug of eDNA). (n=8) **(B)** Crystal violet assay of PAO1*ΔwspF* hydrated biofilm at 24h treated with 1µl of PAO1 EPS (2ug of eDNA) (n=8). **(C)** Electron plasma resonance spectroscopy of the genomic DNA isolated from PAO1 and PAO1*ΔwspF*. (left panel) before DNaseI digestion and (right panel) after DNaseI digestion. **(D)** The growth curve of PAO1 after treatment with intact genomic DNA ($_i$DNA) and fragmented genomic DNA ($_f$DNA) isolated from PAO1. **(E)** The digital photomicrograph of the PAO1 biofilm at 12h treated with intact genomic DNA ($_i$DNA) and fragmented genomic DNA ($_f$DNA). **(F)** The Pearson's coefficient from fig 4B of the PAO1 biofilm at 12h treated with intact genomic DNA ($_i$DNA) and fragmented genomic DNA ($_f$DNA) were plotted graphically as mean ± SD (n=4).

Figure S6

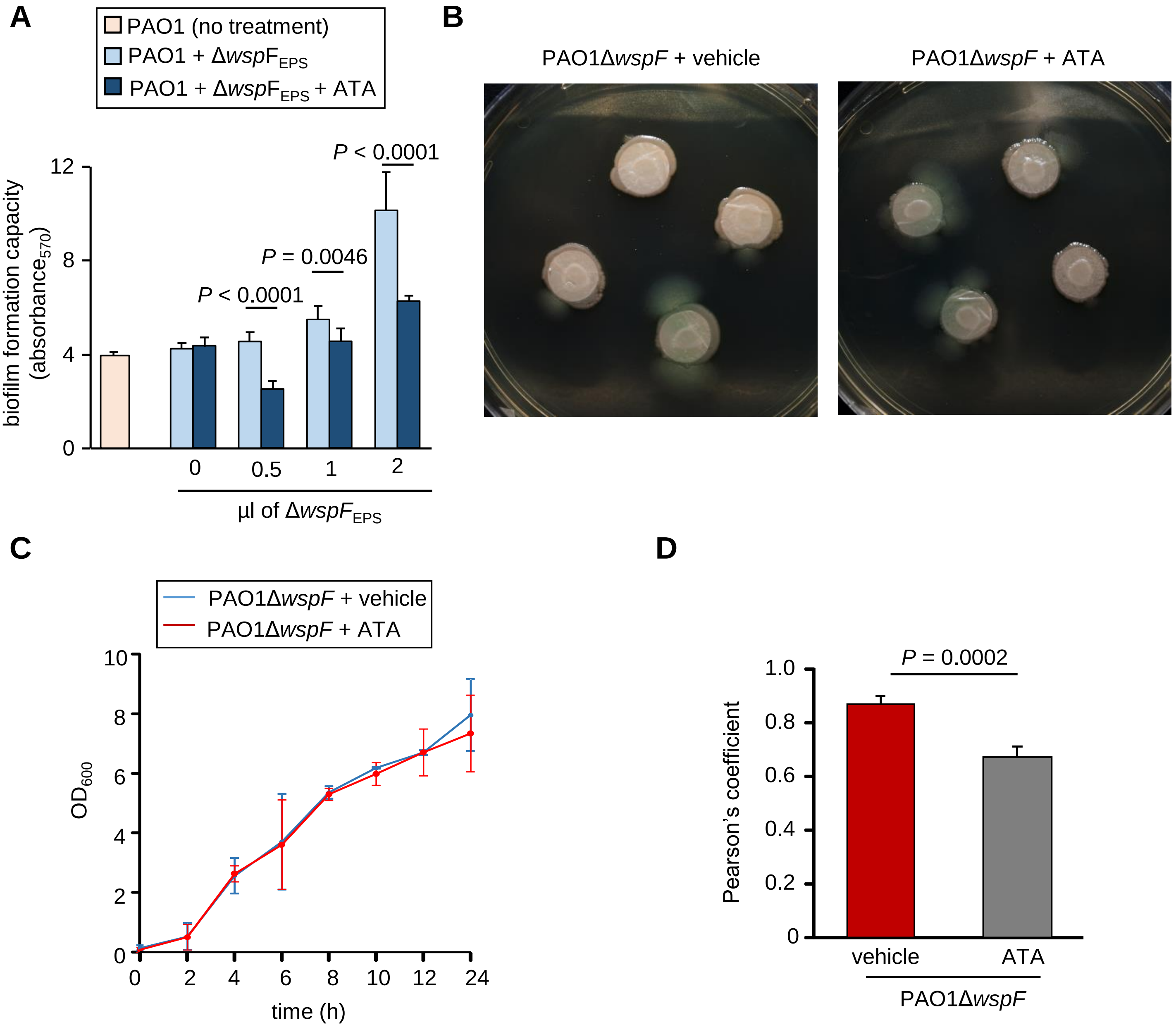

Supplementary Figure 6. Effect of ATA on PAO1Δ*wspF* biofilm. Related to Figure 4D-F. (**A**) Crystal violet assay of untreated PAO1 hydrated biofilm at 12h and PAO1 hydrated biofilm treated with different volume of PAO1*ΔwspF* EPS (1µl = 2ug of eDNA) in presence of 0.5µM of ATA (n=8). Inhibition of DNA–protein interaction compromised *in vitro* PAO1 biofilm formation. (**B**) The digital photomicrograph of the PAO1Δ*wspF* biofilm at 24h treated with buffer and ATA. (**C**) The growth curve of PAO1Δ*wspF* biofilm treated with buffer and ATA. (**D**) The Pearson's coefficient of the PAO1Δ*wspF* biofilm at 24h treated with vehicle and ATA were plotted graphically as mean ± SD (n=4).

Transparent Methods

Bacterial strain. *P. aeruginosa* prototypical strain PAO1 and its isogenic RSCV PAO1Δ*wsp*F were used in this study. Under laboratory conditions, emergence of RSCVs relies on loss-of-function mutations in the methylesterase-encoding gene *wspF* (Pu et al., 2018)**.** Cultures were routinely grown on Luria-Bertani (LB) agar or in LB broth.

***In vitro* biofilm.** *In vitro* PAO1 and PAO1Δ*wsp*F biofilm were developed on a 10 mm polycarbonate membrane (PCM) filter as described previously (Banerjee et al., 2015). Briefly, following overnight culture in LB medium at 37°C, the bacteria were inoculated on sterile PCM filters placed on trypticase soy agar (TSA) (Catalog No: 22091, Sigma-Aldrich, USA) plates. The plates were incubated at 37°C for 24h, after which the PCMs were transferred to a new TSA agar plate. The PCM filters were kept for additional 24h for the biofilm to mature.

Treatment of *in vitro* biofilm. In some experiments, the 48h matured biofilm was treated with RNase free DNaseI (Roche, 04716728001) for 30 min at 37°C prior to sample processing. The 1X buffer (Roche, 04716728001) without the DNaseI (Roche, 04716728001) was used as vehicle control. In other set of experiments, we treated the 48h biofilm cultures with 0.5µM ATA (aurintricarboxylic acid) (A1895 Sigma) for 30 min at 37°C. For PAO1 and PAO1Δ*wsp*F biofilm assays, a time point of 12h and 24h was chosen.

Bacterial growth curve. *P. aeruginosa* PAO1 and PAO1Δ*wsp*F were cultured in Luria-Burtani (LB) medium at 37°C in round bottom tubes with continuous shaking at 300 rpm. The optical density of the media at 600 nm was recorded over different time points with or without treatments and plotted graphically.

Scanning transmission electron microscopy (STEM) sample preparation. Biofilms were primarily fixed with 2.5% glutaraldehyde and 2% paraformaldehyde in 0.15M-cacodylate buffer. After washed three times with 0.15M-cacodylate buffer, the primarily fixed biofilms were post-fixed with 2% reduced osmium tetroxide. The biofilms were then washed with distilled water and further stained with 1% uranyl acetate. The stained samples were dehydrated in an increasing series of ethanol (30%, 50%, 70%, 80%, 90%, 2×100%) for 15 min each. After dehydration, samples were immersed in 1:0, 3:1, 1:1 and 1:3 acetone/resin for 60 minutes each and then kept in 100% resin overnight. Lastly the samples were transferred in fresh 100% resin and incubated at 65°C for 2 days to form a polymerized resin block.

90nm ultra-fine sections were cut from the resin block using a Reichert-Jung (Leica, Wetzlar, Germany) Ultracut E ultramicrotome. The thin sections were picked up with a loop and put on 400 meshes copper grids. For tomography, 500 nm thick sections were cut and put on copper grids with parallel bars. The thick sections were oriented so that the biofilm-growing base was perpendicular to the parallel bar. The copper grids with resin-embedded samples were air dried and then coated with 3nm thick amorphous carbon on both sides.

STEM image acquisition. Electron micrographs were collected in STEM mode on a Tecnai F20 S/TEM (Thermo Fisher Scientific, Hillsboro) with high angle angular dark field (HAADF) detector. Microscope was operated at an acceleration voltage of 200kV using Tecani Imaging and Analysis (TIA) software. Images size was 2,048×2,048 pixels. Exposure time was 25s.

STEM Tomography and data processing. STEM tomography was collected on the FEI probe-corrected Titan3™ 80-300 S/TEM (Thermo Fisher Scientific, Hillsboro). The microscope was operated at an acceleration voltage of 300kV. Images with 2,048×2,048 pixels were recorded with HAADF detector. Single-axis tilt series ranging from -65° to 65° with 1° interval steps were recorded by using the FEI Xplore3D software (Movies S7-8). Sample tilting, focusing and image shift correction were controlled by Xplore3D software. STEM dynamic focus was activated to ensure areas of interest are imaged in focus even at high tilt angles. Tracking was set after exposure. Tomographic tilt series were aligned and reconstructed using IMOD software package (University of Colorado). 3D reconstruction was built by weighted back-projection

method. Images were visualized using IMOD, Chimera and Avizo software's. Movies were made using Avizo software.

Scanning electron microscopy. Scanning electron microscopy was performed on the *in vitro* biofilm as described previously (Banerjee et al., 2015). Briefly, the biofilm on PCM filters were fixed in 4% formaldehyde / 2% glutaraldehyde solution for 48 hours at 4°C, and subsequently dehydrated in graded ethanol series. The samples were mounted on an aluminum stub and were sputter coated with gold-palladium (Au/Pd) and imaged under the scanning electron microscope (XL 30S; FEG, FEI Co., Hillsboro, OR) operating at 5 kV in the secondary electron mode.

Density gradient centrifugation of *in vitro* biofilm of PAO1 and PAO1Δ*wsp*F. 48h i*n vitro* biofilm of PAO1 and PAO1Δ*wsp*F were gently vortexed in 1 ml sterile PBS for 30s to make homogenous mixture. 20 ml Ficoll (Ficoll® Paque Plus, GE17-1440-03 SIGMA) was taken in a 50 ml centrifuge tube. The bacterial suspension was slowly poured on the Ficoll and the tube was centrifuged at 1800 g for 20 min. The supernatant and pellet were taken separately in new tubes. The supernatant was centrifuged at 12,000 g for 10 min at 4°C to collect the bacteria. Bacteria obtained from both supernatant and pellets were washed three times with sterile PBS. The bacterial pellet was then immediately processed for protein isolation. The total protein concentration was quantitated using BCA assay (Pierce, # 23228).

Fluorescence staining of biofilm and confocal microscopy: Biofilms were washed three times with sterile PBS. The density and architecture of the extracellular polymeric substances (EPS), referred to here as "extracellular matrix," was stained with 100 mg/ml FITC-conjugated Hippeastrum Hybrid Amaryllis lectins. (HHA; specific for Psl) for two hours at 4°C (Baker et al., 2015). The biofilms were then washed and fixed with 4% paraformaldehyde. Prior to imaging, the biofilms were stained with DAPI. For detection of extracellular DNA, TOTO™-1 iodide staining (ThermoFisher Scientific, Cat # T3600; dilution 1:1000) was done(Turnbull et al., 2016). Confocal microscopy was performed using Olympus FV1000 filter confocal system at 40x, N.A. 0.45 objective lens (Olympus America Inc, Melville NY). Live cell imaging was done with LSM880 laser scanning confocal microscope.

For the live dead staining of the bacteria, 48h biofilms were incubated for 30 min with a solution containing Syto Green (live) and propidium iodide (dead) (Invitrogen) as per manufacture's instruction. For the study of biofilm matrix, 48h biofilms were incubated for 45min with a solution containing Film Tracer SYPRO Ruby dye (Invitrogen) as previously described (Yi et al., 2011), with minor modifications. SYPRO Ruby fluorescence images were acquired by Olympus FV1000 filter confocal microscope with excitation at 457nm and emission at 610nm. After z-series acquisition, a z image through the image stack, perpendicular to the substrate, was generated.

Bacterial oxygen consumption assay. The XFe96 Extracellular Flux Analyzer (Seahorse Bioscience) was used to quantitate oxygen consumption rates (OCRs) as described previously. Briefly, 48h after biofilm were disrupted and separated using density gradient centrifugation. The separated fractions were diluted to an OD_{600} of ~0.3. Cells were added to XF Cell Culture Microplates pre-coated with poly-D-lysine (PDL). Cells were centrifuged for 10 min at 1,400 × g in a Multifuge ×1R (M-20 rotor) to attach them to the pre-coated plates. After centrifugation, 160μL of fresh media was added to each well.

Extracellular Polymeric Substance (EPS) isolation: EPS was isolated and purified from *in vitro* biofilm as mentioned by Bales et al. with some modifications (Bales et al., 2013). Briefly, 48h old *in vitro* biofilm was transferred into 500μL of PBS (phosphate buffered saline), and vortexed. Complete recovery of EPS was done by vortexing at least for three times. PCM membrane was discarded after recovery of EPS. 37.5% of formaldehyde was added into the cultured solution and incubated for 1 hour at room temperature on shaker (100 rpm). The treated solution was mixed with 1M sodium hydroxide and incubated for 3h at room temperature. This solution was centrifuged at 16,800g for 1 hour at 40°C. Supernatant was filtered through 0.2μm filter. EPS was stored at -80°C for further use. Sterility of purified EPS was checked by spreading 50μL of EPS on TSA agar plates followed by incubation at 37°C for 48 hours. For normalization, the total polysaccharides in EPS was measured by total carbohydrate assay kit (Sigma Aldrich, USA) following manufacture's instruction. The whole EPS was electrophoresed on 1% agarose gel for visualizing the EPS

DNA. In some experiments, the DNA was extracted from EPS using GenElute™ miniprep binding column (Sigma-Aldrich, USA) and subjected to EPRS analysis using Agilent high sensitivity D1000 tape station.

DNase assay of EPS. The DNase activity of the EPS isolated from PAO1 and PAO1Δ*wsp*F biofilm were measured using the DNaseAlert™ QC System (Invitrogen) as per manufacturer's protocol.

Crystal violet assay for biofilm quantification. *P. aeruginosa* (PAO1 and PAO1Δ*wsp*F) were cultured in Luria-Bertani (LB) medium at 37°C in pre-sterilized 96 well flat bottom polystyrene micro-titre plates in triplicates as described previously (O'Toole, 2011) with modifications. Briefly, old media was discarded, and biofilm was washed three times with PBS and air-dried. Biofilm was stained with 200 µl of crystal violet solution (Fisher Scientific, S25275B) (0.1%) and incubated at 37°C for 15 minutes. The excess crystal violet was removed from wells and washed three times with PBS. 200µl of 70% ethyl alcohol was added in each well. Plate was incubated at 37°C for 10 minutes. Biofilm growth was monitored in terms of $O.D_{570}$ nm using micro plate reader.

Genomic DNA isolation and agarose gel electrophoresis. Genomic DNA from PAO1 and PAO1Δ*wsp*F was isolated by GenElute™ Bacterial Genomic DNA Kit (Sigma-Aldrich, USA) following manufacturer's instructions. 1.5 mL of 10^6 CFU mL^{-1} logarithmic bacterial broth culture was taken for genomic DNA isolation. The bacterial cells were pelleted by centrifuging the tube at 12,000-16,000 g for 2 min. The pellet was resuspended in 180µL of lysis solution followed by gentle vortex. 20µL of RNase A was added to the solution and incubated for 2 min at room temperature. 20µL of proteinase K was added to the solution and incubated at 55°C for 30 min. 500µL of column preparation solution was added to each column and centrifuged at 12000 g for 1 minute. 200µL of ethanol was added to the cell lysate and mixed by vortexing for 10 s. The entire solution was transferred into the column and centrifuged at 6500 g for 1 min. The flow though was discarded, and the column was rinsed with 500µL of wash solution 1. The column was further washed with wash solution and centrifuged at 12000-16000 g for 3 min. 200µL of elution buffer was added to the column and incubated for 5 min at room temperature. Genomic DNA was eluted following centrifugation of the column at 6500 g for 1 min. Further the genomic DNA was visualized on 0.8% agarose gel and analyzed by EPRS using Agilent genomic DNA tape station.

Next Generation Sequencing: PAO1 and PAO1Δ*wsp*F EPS DNA samples were isolated and quality check was performed by Qubit DNA Assay Kit. All samples passed internal quality control. The samples were subjected to fragmentation, adaptor addition, with final QC by Agilent 2100 Bioanalyzer and real-time PCR quantification. Whole Genome Sequencing (8 Million reads, 2x75bp, PE) was performed. The reads were first trimmed for adaptor sequences and error corrected. Genome assembly was performed using SPAdes. Genomic DNA of PAO1 and PAO1Δ*wsp*F were also sequenced and compared with PAO1 reference sequence (accession number: NC_002516) showing high synteny with the reference sequence (Figure S1,2), indicating the assembly quality was adequate for subsequent analysis. Coverage analysis of each genomic region was performed. The average coverage for each EPS DNA was found to be around 300x. Read coverage was then compared between PAO1 EPS and PAO1Δ*wsp*F EPS sample. Figure 3C shows sorted alignment with PAO1 reference sequence

DNA digestion. The genomic DNA isolated from PAO1 and PAO1Δ*wsp*F strains were subjected to DNA digestion using RNase free DNaseI (Roche, 04716728001) for 30 min at 37°C. The DNA was purified to remove the DNaseI and 500 ng of either this digested DNA or intact DNA (without DNaseI treatment and purification) was added to the bacterial culture on PCM.

Statistical analysis. Samples were coded and data analysis was performed in a blinded fashion. Data were reported as mean ± SD. All experiments were performed at least three times. Student's t test (two-tailed) was used to determine significant differences. Comparisons among multiple groups were tested using analysis of variance (ANOVA). $p<0.05$ was considered statistically significant.

Supplemental References

Baker, P., Whitfield, G.B., Hill, P.J., Little, D.J., Pestrak, M.J., Robinson, H., Wozniak, D.J., and Howell, P.L. (2015). Characterization of the Pseudomonas aeruginosa Glycoside Hydrolase PslG Reveals That Its Levels Are Critical for Psl Polysaccharide Biosynthesis and Biofilm Formation. J Biol Chem *290*, 28374-28387.

Bales, P.M., Renke, E.M., May, S.L., Shen, Y., and Nelson, D.C. (2013). Purification and Characterization of Biofilm-Associated EPS Exopolysaccharides from ESKAPE Organisms and Other Pathogens. PLoS One *8*, e67950.

Banerjee, J., Ghatak, P.D., Roy, S., Khanna, S., Hemann, C., Deng, B., Das, A., Zweier, J.L., Wozniak, D., and Sen, C.K. (2015). Silver-Zinc Redox-Coupled Electroceutical Wound Dressing Disrupts Bacterial Biofilm. PLoS ONE *10*, e0119531.

O'Toole, G.A. (2011). Microtiter Dish Biofilm Formation Assay. Journal of Visualized Experiments : JoVE, 2437.

Pu, M., Sheng, L., Song, S., Gong, T., and Wood, T.K. (2018). Serine Hydroxymethyltransferase ShrA (PA2444) Controls Rugose Small-Colony Variant Formation in Pseudomonas aeruginosa. Frontiers in Microbiology *9*, 315.

Turnbull, L., Toyofuku, M., Hynen, A.L., Kurosawa, M., Pessi, G., Petty, N.K., Osvath, S.R., Cárcamo-Oyarce, G., Gloag, E.S., Shimoni, R.*, et al.* (2016). Explosive cell lysis as a mechanism for the biogenesis of bacterial membrane vesicles and biofilms. Nature communications *7*.

Yi, S., Sahni, N., Daniels, K.J., Lu, K.L., Srikantha, T., Huang, G., Garnaas, A.M., and Soll, D.R. (2011). Alternative mating type configurations (a/alpha versus a/a or alpha/alpha) of Candida albicans result in alternative biofilms regulated by different pathways. PLoS biology *9*, e1001117.
